# Artificial Heart Rejects High Tech? Lessens Learnt from Non-pulsatile VAD with Straight Impeller Vanes

**DOI:** 10.2174/1874120700701010035

**Published:** 2007-07-17

**Authors:** Kun-xi Qian

**Affiliations:** Jiangsu University, Biomedical Engineering Institute, Zhenjiang, 212013, China

## Abstract

Despite the progresses in developing pulsatile impeller pump and impeller total heart, as well as in applying streamlined impeller vanes, the best results in application of artificial heart pumps have been achieved by nonpulsatile univentricular assist pump with straight impeller vanes until now. It seems all efforts and successes have been done in vain because artificial heart rejects Hi-Tech! This paper recalls some important achievements in R&D of artificial heart in past 25 years and shares author’s experiences with the readers.

## PULSATILE OR NONPULSATILE FLOW

In the early 1980s, people did not know whether a nonpulsatile pump could support the circulation partly or totally. Most medical doctors preferred to use pulsatile flow pumps, but the serious problems resulted in the application of diaphragm pumps forced the researchers to change the pumping principle. It followed thereafter two tendencies that some one tried to maintain the animal circulation with nonpulsatile flow as long as possible and others made efforts to develop a pulsatile rotary pump. The conflict of nonpulsatile or pulsatile perfusion lasted to the end of 1990s. As the author published a pulsatile impeller pump with low hemolysis (Artificial Organs, 1989 / 13 / 02 P 162-169), the nonpulsatile pump could make the animal experimental survival only few weeks. Then the author received over 20 international invitations for giving lectures, sending reprints or writing new papers. In the following 10 years, however, nonpulsatile pump achieved long-term survival in animal experiments worldwide [1], and now a patient has lived by a nonpulsatile pump for about 7 years [2]. Thereby, nobody concerns pulsatile rotary pump any more.

**Fig. (1):**
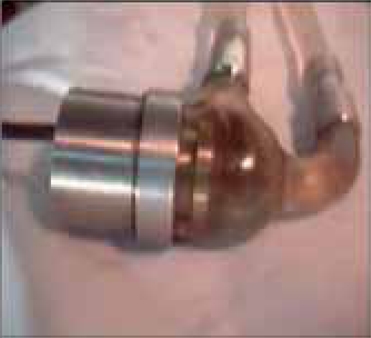
Pulsatile impeller pump changes its rotating speed periodically to produce flow waves and thereby its rotor reciprocates axially under action of hydraulic force and magnetic force in motor.

The pulsatile impeller pump is shown in (Fig. **1**). The pulsatility of blood flow and pressure is achieved by changing the rotating speed periodically. To avoid huge crash of blood elements during few micro seconds by velocity increase, the impeller vanes are twisted for blood cells to escape out from the impeller axially, thus the flow and pressure increase but the shear stress varies unremarkably. The rotor reciprocates axially under the hemo-dynamic force and magnetic force in motor: During ejecting blood, the hemodynamic force is larger than magnetic force and the rotor will move to inlet; during diastole, the rotor will return to its original position by magnetic force.

## IMPELLER ASSIST OR TOTAL HEART

**Fig. (2):**
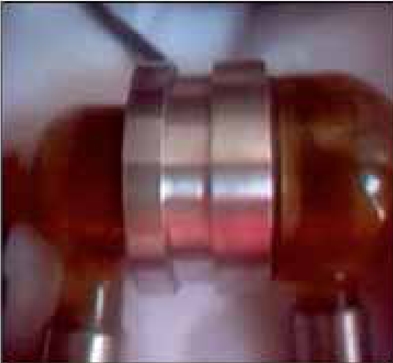
Impeller total heart has one motor, driving two pumps, which support both systemic and pulmonary circulations of patients or animals with pulsatile flow.

The R&D of artificial heart has 50 years history. In the first 25 years, diaphragm pumps imitating the natural heart displacement function played a leading role; the total hearts were used in animal experiments mostly. From the beginning of 1980s, the attention had concentrated more and more on rotary pumps, the interest was held still in total heart. As the author presented an impeller total heart at The 33^rd^ Conference of ASAIO in New York, many investigators took this prototype design as “representing the developing direction of heart pumps.” Dr. Kolff, the Father of artificial heart, wrote excitedly a brief note to me: “investigating invitation to S.L.C.” Then a related paper was published in Proceeding of the Conference (Trans. ASAIO, 1987 / 33 / 03 P 704-707). This device is shown in (Fig. **2**). It has a DC brushless motor driving two pumps, can support systemic and pulmonary circulations meanwhile. Thus it was called either impeller total heart or biventricular assist impeller pump. Furthermore, both the pumps produce pulsatile flow synchronously by changing the rotating speed periodically. This is unique total heart driven by a single motor but ejecting the flow in the left and right pumps simultaneously. The flow balance between the left and right pump can be automatically achieved without need of control, because the impeller pump has a self-modulation property, its flow will increase after the decreasing pressure head or decrease after the increasing pressure head.

As mentioned above, the best results in application of artificial heart pumps have been achieved by nonpulsatile univentricular assist pump. That means the uni-ventricular assist pump can meet the main requirements in most cases. Even in case needing biventricular assists, medical doctors use two single pumps instead of one double pump.

## CURVED OR STRAIGHT IMPELLER VANES

Hmolysis reduction was a heavy point in developing rotary pumps in 1980s. In order to reduce the stress in the pump, the author established an analytical three-dimensional method for impeller design. Three partial differential equations of continuity, motion and energy were solved to obtain the analytical expressions of velocity distribution and stream surfaces or stream lines. Then the vanes were designed according to these stream surfaces (“Progress in impeller pumps in China.” In Assisted Circulation, ed. F. Unger, Springer Verlag Berlin Heidelberg New York.1989 P.197-214). This method had been acknowledged by Dr. Olsen, the successor of Dr. Kolff, he wrote me in 1992 to express his interests in streamlined design of impeller, because he felt that it was “informative” and “convincing”. I’m glad that others have now began to use this idea [3].

The theoretical results have been certified by CFD (J Med Eng Technol. 2006 Nov-Dec; 30 (6):353-7) and PIV (J Med Eng Technol. 2007 Jul-Aug; 31 (4):239-42) modern technologies. Fig. (**3**) exhibits the CFD results that clearly demonstrate that the streamlines are coincide with the vanes of streamlined impeller while in strait vanes the streamlines have crash and separations with vanes.

## DISCUSSIONS

In any scientific project the basic research is always done ahead of the applications, the artificial heart pumps too. It’s understandable if the application indicates that the basic research done previously is not useful, no one will insist the results obtained from the basic research works. If the application could go ahead of the basic research, on the contrary, maybe many efforts made in vain could be avoided. For example, if the nonpulsatile univentricular pump with straight vanes could achieve the good results earlier, no one would spend time and efforts to develop a pulsatile bivebtricular assist pump with streamlined vanes.

In the past 10 years, maglev bearing used in heart pumps has been extensively studied but meanwhile the mechanical bearing heart pump has achieved excellent results [2]. A new question arises: should we use Magnetic or Mechanical Bearing?

## Figures and Tables

**Fig. (3):**
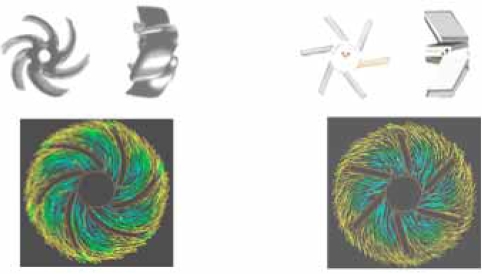
Streamlined impeller vanes (left) and straight impeller vanes (right). Computational Fluid Dynamics demonstrates the flow pattern coincides with the vanes in streamlined impeller but has crash or/and separation in straight vanes.
